# Editorial: Enamel Research: Mechanisms and Characterization

**DOI:** 10.3389/fphys.2016.00374

**Published:** 2016-09-09

**Authors:** Bernhard Ganss, Megan Pugach-Gordon

**Affiliations:** ^1^Mineralized Tissue Lab, Dentistry - Matrix Dynamics Group, University of TorontoToronto, ON, Canada; ^2^Department of Mineralized Tissue Biology, The Forsyth Institute, Harvard School of Dental MedicineCambridge, MA, USA

**Keywords:** enamel maturation, physiology, mineralized tissue, amelogenin, structural biology

The idea to compile the present collection of articles on the topic on dental enamel formation and maturation was born in the course of a discussion over a glass of wine during the 11th International Conference on the Chemistry and Biology of Mineralized Tissues (ICCBMT) in Lake Geneva, Wisconsin in November 2013. Both of us felt that several issues specific to the mineralization of enamel deserved further exploration, and thus motivated, we approached the supportive editorial team at Frontiers. Over the course of the coming months a total of 15 scientists with a passion for enamel agreed to contribute to what is now compiled as an e-book. Although it may seem a long time since its conception, this publication addresses some very important and contemporary aspects of enamel biology and comes as a timely prelude to the upcoming Conferences, *Enamel 9* in Harrogate (UK) in October/November 2016 and the 12th *ICCBMT* in May/June 2017 in Potsdam (Germany).

A number of prominent enamel researchers have made this e-book what it is today. We have somewhat arbitrarily grouped all contributions into three parts. The first part describes and discusses general concepts and limitations of current approaches to understanding enamel biology. Robinson's Introduction provides an excellent historical review of multiple events occurring during the enamel maturation stage and their relationships to enamel dysplasias, while the article by Ganss and Abbarin highlights the role of recently identified proteins beyond the enamel maturation stage. Pugach and Gibson address the benefits and limitations of mouse models to study enamel development, and Goldberg et al. stress the importance of comparative studies between mouse molars and incisors to appreciate the full complexity of enamel development. Sarkar et al. draw comparative conclusions between two of the very few available ameloblast-like cell lines. Babajko et al. describe the role of the transcription factor MSX2 in ameloblasts and highlight its functional analogies with other mineralized tissues.

The second part focuses on individual aspects of enamel proteins and their processing during the formation and mineralization of enamel. The role of specific regions within the most prominent enamel protein, amelogenin, is discussed in two different articles, by Lu et al., as well as Gopinathan et al. Both relate to hydroxyapatite binding properties and the control of biomineralization. Mazumder et al. discuss the functional relevance of interactions between ameloblastin and amelogenin for enamel mineralization. Margolis et al. highlight the role and importance of mineralization inhibitors in enamel formation and maturation. The next two articles, authored by Zhu et al., as well as Bartlett and Simmer, highlight the roles of MMP20 and particularly KLK4 as amelogenin-processing enzymes *in vitro* and *in vivo*.

The third part focuses on recent methodological advances in the analysis of the enamel structure at the nanoscale. Gordon and Joester describe the distribution of magnesium, carbonate and organic material within mature enamel and its possible functional significance, while Bidlack et al. present the use of helium ion microscopy to delineate the three-dimensional organization of enamel mineral and organic matrix. Last, but certainly not least, the article by Lelli et al. provides a translational perspective on toothpaste additives geared toward remineralizing enamel.

This e-book will be most beneficial for researchers in the area of biomineralization, emphasizing biological concepts, technical developments and some remaining controversies. Altogether we believe that this collage of articles focusing on enamel highlights some of the current perspectives, advances, and challenges in this fascinating research area on an extraordinary natural bioceramic material that keeps us all captivated. It is our hope that this publication will contribute to perpetuating this fascination with enamel in students at all levels of education and in various disciplines.

Our special thanks go to all our contributors who have willingly agreed to take on the extra work for this e-book. We also thank the entire editorial team at the Frontiers office who have patiently guided us along the way.

## Author contributions

All authors listed, have made substantial, direct and intellectual contribution to the work, and approved it for publication.

## Funding

Canadian Institutes of Health Research (CIHR) Grant MOP-119310 National Science and Engineering Research Council (NSERC) of Canada Grant RGPIN403292-11.

### Conflict of interest statement

The authors declare that the research was conducted in the absence of any commercial or financial relationships that could be construed as a potential conflict of interest.

